# Robotic Versus Laparoscopic Adrenalectomy for Adrenal Tumors: An Up-to-Date Meta-Analysis on Perioperative Outcomes

**DOI:** 10.3390/cancers17010150

**Published:** 2025-01-05

**Authors:** Giuseppe Esposito, Barbara Mullineris, Giovanni Colli, Serena Curia, Micaela Piccoli

**Affiliations:** Department of General, Emergency Surgery and New Technologies, Baggiovara General Hospital Azienda Ospedaliero Universitaria di Modena, Via Pietro Giardini 1355, 41126 Modena, Italy; mullineris.barbara@aou.mo.it (B.M.); colli.giovanni@aou.mo.it (G.C.); curiaserena@gmail.com (S.C.); piccoli.micaela@aou.mo.it (M.P.)

**Keywords:** adrenalectomy, adrenal gland surgery, robotic adrenalectomy, laparoscopic adrenalectomy, robotic surgery

## Abstract

**Background:** Minimally invasive surgery (MIS) for adrenal glands is becoming increasingly developed worldwide and robotic surgery has advanced significantly. Although there are still concerns about the generalization of outcomes and the cost burden, the robotic platform shows several advantages in overcoming some laparoscopic shortcomings. **Materials and Methods:** A systematic review and meta-analysis were conducted using the PubMed, MEDLINE and Cochrane library databases of published articles comparing RA and LA up to January 2024. The evaluated endpoints were technical and post-operative outcomes. Dichotomous data were calculated using the odds ratio (OR), while continuous data were analyzed usingmean difference (MD) with a 95% confidence interval (95% CI). A random-effects model (REM) was applied. **Results:** By the inclusion of 28 studies, the meta-analysis revealed no statistically significant difference in the rates of intraoperative RBC transfusion, 30-day mortality, intraoperative and overall postoperative complications, re-admission, R1 resection margin and operating time in the RA group compared with the LA. However, the overall cost of hospitalization was significantly higher in the RA group than in the LA group, [MD USD 4101.32, (95% CI 3894.85, 4307.79) *p* < 0.00001]. With respect to the mean intraoperative blood loss, conversion to open surgery rate, time to first flatus and length of hospital stay, the RA group showed slightly statistically significant lower rates than the laparoscopic approach. **Conclusions:** To our knowledge, this is the largest and most recent meta-analysis that makes these comparisons. RA can be considered safe, feasible and comparable to LA in terms of the intraoperative and post-operative outcomes. In the near future, RA could represent a promising complementary approachto LA for benign and small malignant adrenal masses, particularly in high-volume referral centers specializing in robotic surgery. However, further studies are needed to confirm these findings.

## Simple Summary

Laparoscopy represents the gold standard surgical approach for adrenalectomies. Although the robotic platform has been reported to overcome the innate shortcomings of laparoscopic surgery, its use in adrenalectomies remains debated. The aim of this paper is to compare the surgical and postoperative outcomes of robotic adrenalectomy (RA) and laparoscopic adrenalectomy (LA) for adrenal tumors by the inclusion of the largest number of studies. With respect to the mean intraoperative blood loss, conversion to open surgery rate, time to first flatus and length of hospital stay, the RA group showed slightly statistically significant lower rates than the laparoscopic approach. However, the overall costs of hospitalization were significantly higher in the RA group than the LA group. In the near future, RA could represent a promising complementary approach to LA for benign and small malignant adrenal masses, particularly in high-volume referral centers specializing in robotic surgery.

## 1. Introduction

Minimally invasive surgery (MIS) for adrenal glands has been increasingly adopted worldwide thanks to its well-known better perioperative and cosmetic outcomes compared to conventional open approaches. The first laparoscopic adrenalectomy (LA) was described by Gagner et al. in 1992 [1]. Since 2001, LA for benign adrenal tumors has become the gold standard and, in selected cases, LA for small (diameter < 5 cm) malignant or suspected cortical carcinoma represents a safe and feasible treatment [2,3,4]. However, concerns remain regarding the oncologic safety of MIS, particularly in advanced adrenocortical carcinoma (ACC) and metastatic adrenal tumors, which may require multi-organ en bloc resections with extensive lymphadenectomy; therefore, open adrenalectomy remains the treatment of choice for malignant lesions in all relevant guidelines [5]. Laparoscopic adrenal surgery is a technically demanding procedure due to the limited working space, the absence of 3Dvision and the small range of motion of rigid instrumentation [6,7]. In 2001, the first transabdominal lateral fully robotic adrenalectomy (RA) was reported by Horgan et al. [8] and, in 2010, Colvin et al. [9] first described the posterior retroperitoneal RA approach. Although the robotic platform has been reported to overcome the innate shortcomings of laparoscopic surgery by providing a high-definition 3D stereoscopic view with magnification, tremor filtering, and seven degrees of freedom instrument motion, significant improvements in the surgical outcomes of RA over the laparoscopic procedure have yet to be demonstrated and remain debated [10]. However, the robotic approach has been proven to be more advantageous than LA in the posterior retroperitoneal approach and in cortical-sparing adrenalectomy [11,12]. The retroperitoneal technique also shows some technical advantages as it avoids entering the abdominal cavity and mobilizing the nearby structures, which is particularly beneficial for obese patients or those with possible adhesions [13]. Furthermore, in the era of MIS, partial adrenalectomy (PA) has emerged as a valuable and feasible surgical option in the management of small (3–4 cm in diameter) and well-circumscribed adrenal masses, particularly in patients with bilateral disease, hereditary syndromes (e.g., multiple endocrine neoplasia type 2, von Hippel–Lindau syndrome, and familial pheochromocytoma) or unilateral, benign, hormonally active tumors such as aldosterone- or cortisol-producing adenomas. The preservation of endogenous adrenal function has been demonstrated to reduce steroid dependency, thereby improving postoperative outcomes, including a reduction in blood loss, the operating time, and overall complication rates. Despite these advantages, PA remains limited by the need for surgeon expertise and technical challenges. However, the robotic platform has the potential to overcome some of these limitations when compared with the laparoscopic approach for PA [14,15].

However, a previous meta-analysis disclosed contrasting results in terms of the estimated intraoperative blood loss, postoperative morbidity, mortality, conversion rate and operating time between RA and LA [4,16]. Therefore, the primary aim of this meta-analysis is to compare the surgical and postoperative outcomes of RA and LA for adrenal tumors by the inclusion of the largest number of observational studies and randomized controlled trials (RCTs) on this topic from the current literature.

## 2. Methods

### 2.1. Study Design and Selection

Our systematic review and meta-analysis was designed and reported according to the Preferred Reporting Items for Systematic Reviews and Meta-analyses (PRISMA) statement [17], while the authors predetermined the eligibility criteria for the study and PICOS were established in advance of the reviewing process. The PRISMA checklist is reported as Supplementary File S1. Two investigators (E.G. and M.B.) systematically screened the literature using the PubMed, Cochrane library and MEDLINE databases for articles published up to January 2024; querying three databases maximizes the probability of capturing studies, as stated by Goossen et al. [18]. Our electronic search included the words “robotic adrenalectomy”, “laparoscopic adrenalectomy”, “minimally invasive surgery and adrenalectomy” and “suprarenal gland”. The search was limited to English language articles and to humans. The search strategy is provided in Supplementary File S2. All inconsistencies during the data collection, synthesis and analysis were resolved by the consensus of two authors (E.G. and M.B.). The study protocol was depicted prior to the reviewing process and was registered in the PROSPERO International prospective register of systematic reviews (number CRD42024497752).

#### PICOS

(P)opulation: Patients with adrenal tumors, regardless of the histological category, undergoing minimally invasive adrenalectomy.

(I)ntervention: Robotic adrenalectomy, using both the anterior and posterior approach.

(C)omparison: Laparoscopic adrenalectomy, using both the anterior and posterior approach.

(O)utcome: The surgical and postoperative outcomes.

(S)tudies: Randomized controlled clinical trials and observational clinical studies.

### 2.2. Eligibility Criteria

Our systematic review included all retrospective clinical studies and randomized controlled trials that compared robotic adrenalectomy with laparoscopic adrenalectomy for both malignant and benign adrenal tumors, regardless of the surgical approach (anterior versus posterior). Furthermore, no limits in terms of the size and laterality of the adrenal mass were established as additional eligibility criteria. It is worth noting that, for articles published by the same institutions and/or the same authors with a potentially overlapping patient sample, the most recent study was included unless the studies were reporting on different outcomes or on different populations. Case reports, editorials, reviews, letters and animal studies were excluded. Furthermore, papers involving partial adrenalectomy or pediatric samples were not included. The present meta-analysis did not require Institutional Review Ethical approval or written consent.

### 2.3. Outcomes

The analyzed outcomes were the operating time, the estimated intraoperative blood loss and transfusion rate, the rates of overall intraoperative complications, and conversion to open surgery. As for the post-operative outcomes, the rates of overall post-operative complications and major complications according to Clavien–Dindo ≥ III, the time to first flatus, the length of hospital stay, and the rates of 30-day readmission and 90-day mortality were evaluated. Secondary outcomes were the R1 resection margin rates and the overall cost of hospitalization.

### 2.4. Quality Assessment

The quality of all retrospective included studies was assessed using the Methodological Index for Non-Randomized Studies (MINORS) [19]. This is a quality assessment tool designed to estimate the methodological adequacy of non-randomized studies, and accordingly it was not evaluated for the two RCTs included in the analysis.

### 2.5. Statistical Analysis and Risk of Bias

The statistical meta-analysis was performed using the software Review Manager (RevMan) [Version 5.1. Copenhagen: The Nordic Cochrane Centre, The Cochrane Collaboration, 2011]. The results of the meta-analysis are presented as odds ratios (ORs) with a 95% confidence interval (CI) by using the Mantel–Haenszel method for dichotomous outcomes, and as the mean difference (MD) with a 95% CI by using the generic inverse variance method for continuous variables. The mean and standard deviation (SD) for continuous data, if not available, were estimated from the available median and range using the method described by Hozo et al. [20]. However, for continuous data provided as a median and interquartile range, the mean and SD were estimated by employing the method illustrated by Luo et al. [21] and Wan et al. [22], respectively. The cut-off for statistical significance was set at *p* ≤ 0.05. Heterogeneities between the included trials were tested using Q statistics and the total variation across studies was estimated by I^2^. Accordingly, the severity of heterogeneity and the strength of evidence were interpreted using previously established thresholds: 0–40%: likely minimal; 30–60%: likely moderate; 50–90%: likely substantial; and 75–100%: likely considerable [23]. Moreover, due to the innate clinical and conceptual heterogeneity of the included studies, a random-effects model (REM) was applied, according to the DerSimonian–Laird method. The methodological quality of the distribution and the risk of bias of the included studies were assessed using the Newcastle–Ottawa Scale (NOS) for the 26 observational studies and the RoB 2 tool was used for the 2 RCTs, according to the Cochrane recommendations [24]. The detailed risk of bias assessment with figures and tables is provided in Supplementary File S3. Moreover, the publication bias of the included studies was tested and the funnel plots for each outcome are provided in Supplementary File S3.

## 3. Results

### 3.1. Studies and Patient Characteristics

**Figure 1:**
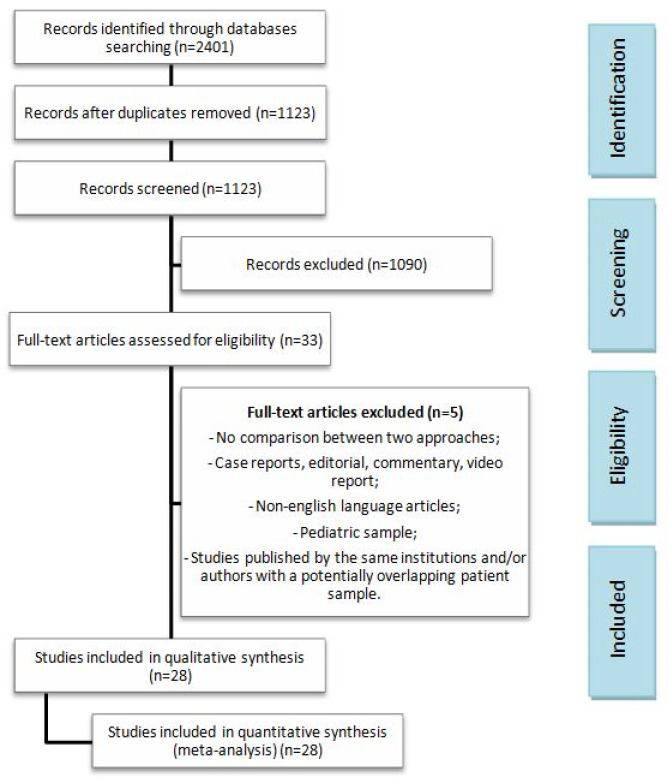
PRISMA flow diagram.

The initial search strategy retrieved 2401 publications concerning minimally invasive adrenalectomy. After the screening of all titles and abstracts and the removal of duplicates, a total of thirty-three full-text articles were retrieved, of which five were excluded because of missing inclusion criteria. The search process with the missing inclusion criteria is presented according to the PRISMA flow diagram in Figure 1. Twenty-six retrospective observational studies [7,9,13,25,26,27,28,29,30,31,32,33,34,35,36,37,38,39,40,41,42,43,44,45,46,47] and two RCTs [48,49] were finally included in our systematic review, involving 4140 patients. Among them, 1446 patients underwent RA, whereas 2694 were offered LA. The baseline features, the quality assessment of the studies included, and the patients’ characteristics are shown in Table 1 and Table 2. The analyzed outcomes are tabulated in Table 3. The two groups were similar regarding their follow-up time, age, body mass index (BMI) and tumor diameter on explant pathology. Interestingly, the American Society of Anesthesiologists’ (ASA) score ≥ III rate was higher in the RA group and the mean follow-up time was longer in the LA group. Moreover, an anterior approach was more frequently performed in the robotic subgroup with a lower rate of malignant histology tumors compared with the LA group. The number of patients in each study ranged from 5 to 367. According to the MINOR scale, the overall quality assessment showed that the included retrospective studies were methodologically adequate, with low heterogeneity regarding their quality, providing a mean score of 22.2 (SD: 0.69) and a median score of 22 (range 20–23) (Table 1).

### 3.2. Surgical Outcomes

#### 3.2.1. Operating Time

**Figure 2:**
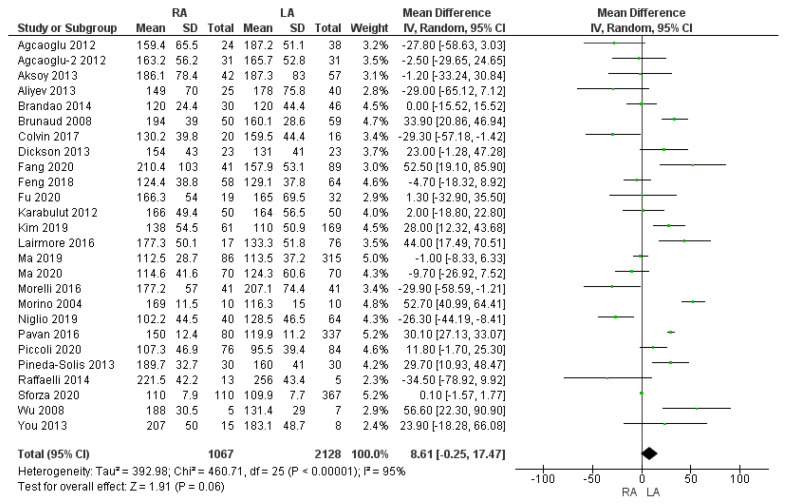
Operating time (min) [5,7,9,13,16,25,27,28,29,30,31,32,33,35,36,37,38,39,40,41,43,45,46,47,48,49].

Twenty-six studies reported the operating time. The mean operating time was 157.2 min in the RA group and 149.6 min in the LA group. The operating time was slightly shorter in the laparoscopic adrenalectomy group; however, the meta-analysis did not show a statistically significant difference [MD 8.61, (95% CI −0.25, 17.47) *p* = 0.06], as shown in Figure 2.

#### 3.2.2. Intraoperative Blood Loss and Intraoperative Red Blood Cell (RBC) Transfusion Rate

**Figure 3:**
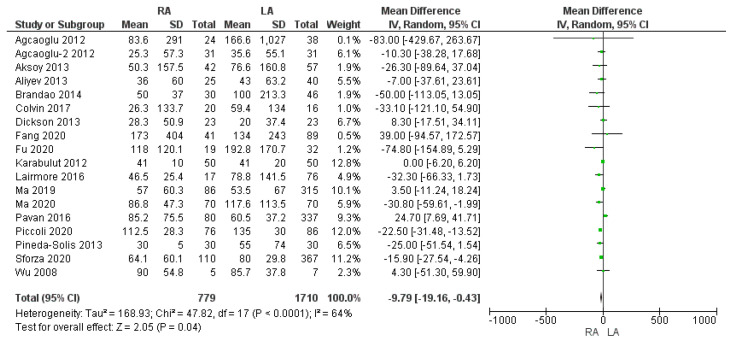
Intraoperative blood loss (mL) [5,7,9,13,27,28,29,30,31,32,33,35,37,41,45,46,47,49].

**Figure 4:**
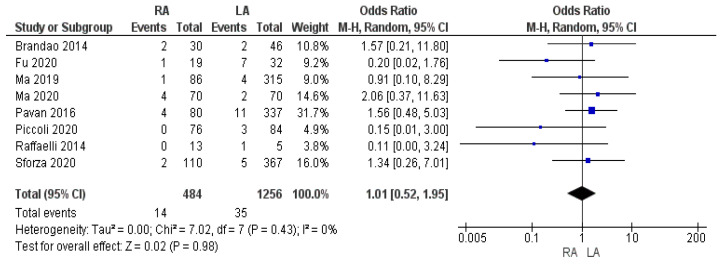
Intraoperative Red Blood Cell (RBC) transfusion rate [7,35,37,38,41,46,47,49].

Our meta-analysis revealed statistically significant slightly increased amounts of estimated intraoperative blood loss in the LA group when compared with the robotic one [MD −9.79, (95% CI −19.16, −0.43) *p* = 0.04], as shown in Figure 3. The mean intraoperative blood loss in the RA and LA groups was 66.9 cc and 85.3 cc, respectively. The mean intraoperative RBC transfusion rate was similar between the two approaches, being 2.9% in the RA group and 2.8% in the LA group. The meta-analysis of this variable was not found to be statistically significant [OR 1.01, (95% CI 0.52, 1.95) *p* = 0.98], as shown in Figure 4.

#### 3.2.3. Conversion to Open Surgery Rate

**Figure 5:**
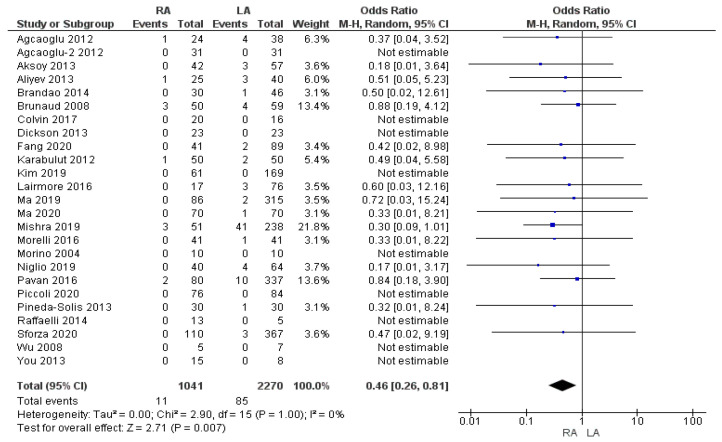
Conversion to open surgery rate [5,7,9,13,16,25,27,28,29,30,31,32,33,35,36,37,38,39,41,42,43,45,48,49].

The reoperation rate was 1.05% (11/1041) in the RA group and 3.7% (85/2270) in the LA group. The meta-analysis showed a statistically significant difference in the rate of conversion to open surgery between the two groups, being higher in the LA group than the RA group [OR 0.46, (95% CI 0.26, 0.81) *p* = 0.007], as shown in Figure 5.

#### 3.2.4. Intraoperative Complication Rate

**Figure 6:**
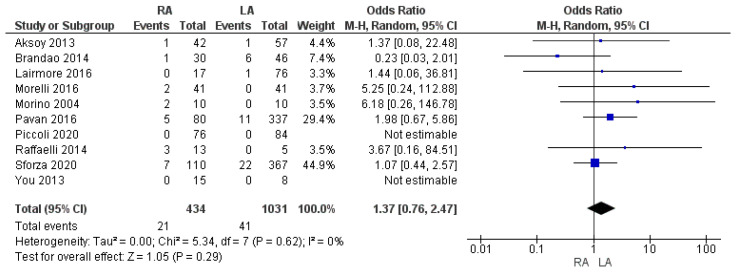
Intraoperative complication rate [7,13,16,33,35,36,37,38,47,48].

The intraoperative complication rate, such as intraoperative hypertension after the removal of the adrenal masses, accidental inferior vena cava damage, spleen injury or bowel perforation, was 4.8% (21/434) in the RA group and 3.9% (41/1031) in the LA group, being slightly higher in the former group. Meta-analysis of the 10 trials did not show a statistically significant difference in the rate of intraoperative complications between the two approaches [OR 1.37, (95% CI 0.76, 2.47) *p* = 0.29], as shown in Figure 6.

### 3.3. Postoperative Outcomes

#### 3.3.1. Time to First Flatus

**Figure 7:**
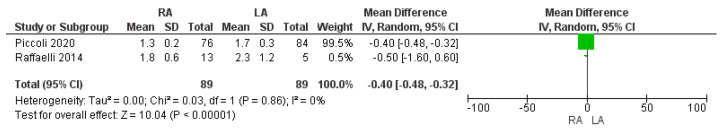
Time to first flatus [7,38].

The mean time to first flatus after surgery was 1.5 days in the RA group and 2 days in the LA group, being shorter for the robotic approach. Only two studies reported this outcome. Our meta-analysis found that the mean difference between the two groups was statistically significant [MD −0.40, (95% CI −0.48, −0.32) *p* < 0.00001], as shown in Figure 7.

#### 3.3.2. Overall Complication Rate

**Figure 8:**
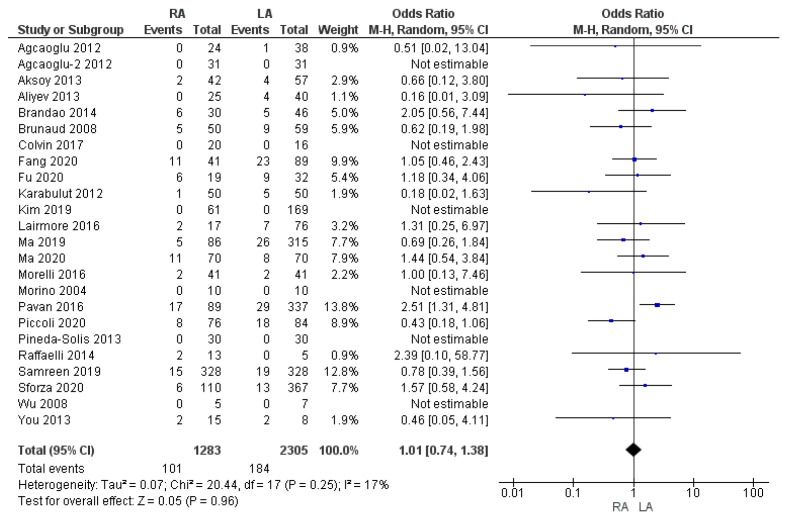
Overall complication rate [5,7,9,13,16,25,27,28,29,30,32,33,35,36,37,38,39,41,44,45,46,47,48,49].

Twenty-four studies assessed postoperative complications. Morbidity (e.g., deep vein thrombosis, wound infection, fever and pulmonary embolism) in the RA group was similar to the LA group: 7.8% (101/1283) and 8% (184/2305), respectively. Our meta-analysis showed that the difference was not statistically significant [OR 1.01, (95% CI 0.74, 1.38) *p* = 0.96], as shown in Figure 8.

#### 3.3.3. Complications Rate According to the Clavien–Dindo ≥ III

**Figure 9:**
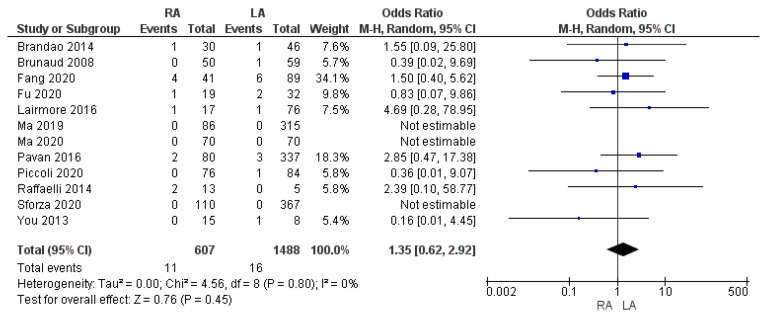
Clavien–Dindo ≥ III complication rate [7,25,33,35,36,37,38,41,45,46,47,49].

Twelve studies reported the frequency of surgical complications according to the Clavien–Dindo classification system. Our meta-analysis focused on the rate of surgical complications, which corresponds to a Clavien–Dindo rating greater than Grade III (Grade ≥ III) between the RA and LA groups. This rate was similar between the two samples: 1.8% (11/607) and 1.1% (16/1488) [OR 1.35, (95% CI 0.62, 2.92) *p* = 0.45], as shown in Figure 9.

#### 3.3.4. Length of Hospital Stay

**Figure 10:**
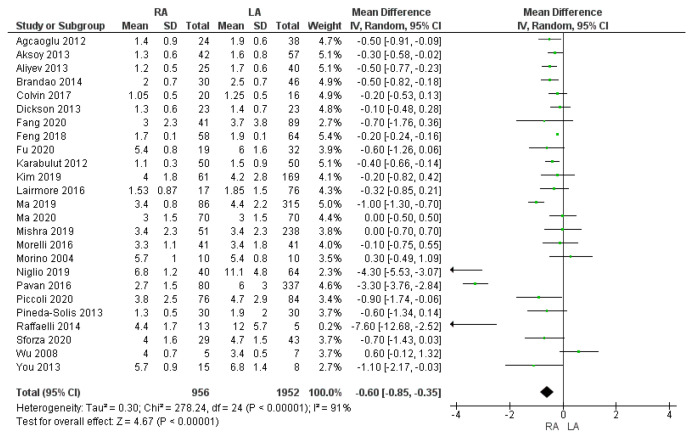
Length of hospital stay [5,7,9,13,16,28,29,30,31,32,33,35,36,37,38,39,40,41,42,43,45,46,47,48,49].

The mean length of hospital stay was 3.06 days in the RA group and 4 days in the LA group. The meta-analysis showed that the hospital stay was 1day longer in the LA group than in the robotic group, and this difference was statistically significant [MD −0.60, (95% CI −0.85, −0.35) *p* < 0.00001], as shown in Figure 10.

#### 3.3.5. Re-Admission to Hospital Rate

**Figure 11:**
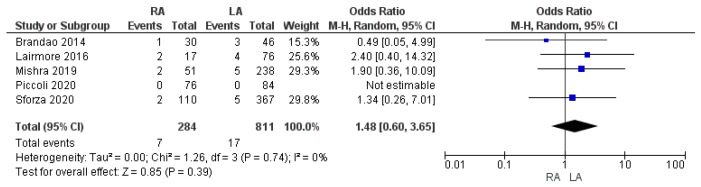
Readmission rate [7,33,37,42,47].

Five studies reported the re-admission to hospital rate. The re-admission rate was 2.5% (7/284) in the RA group and 2.1% (17/811) in the LA group. The meta-analysis of this variable was not found to be statistically significant [OR 1.48, (95% CI 0.60, 3.65) *p* = 0.39], as shown in Figure 11.

#### 3.3.6. R1 Resection Margin Rate

**Figure 12:**
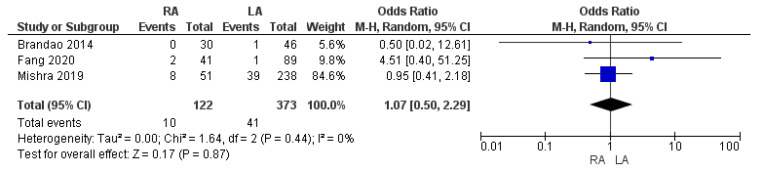
R1 resection margin rate [37,42,45].

A total of 51 patients showed a R1 resection margin on final pathology. The R1 resection margin rate in the RA group was lower than that in the LA group: 8.2% (10/122) and 11% (41/373), respectively. However, the meta-analysis did not show a significant difference in the rate of positive margins between the two groups [OR 1.07, (95% CI 0.50, 2.29) *p* = 0.87], as shown in Figure 12.

#### 3.3.7. Thirty-Day Mortality Rate

**Figure 13:**
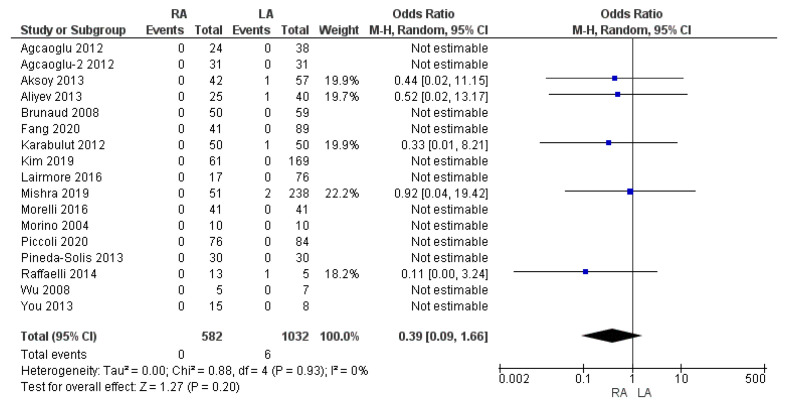
Thirty-day mortality rate [5,7,13,16,25,27,28,29,30,32,33,36,38,39,42,45,48].

Seventeen papers reported the 30-day mortality rate. The mortality rate was 0% (0/582) in the robotic adrenalectomy group and 0.6% (8/1032) in the LA group. The meta-analysis found that this variable was not significantly increased in the laparoscopic group compared with the robotic group [OR 0.39, (95% CI 0.09, 1.66) *p* = 0.20], as shown in Figure 13.

#### 3.3.8. Cost of the Hospitalization

**Figure 14:**
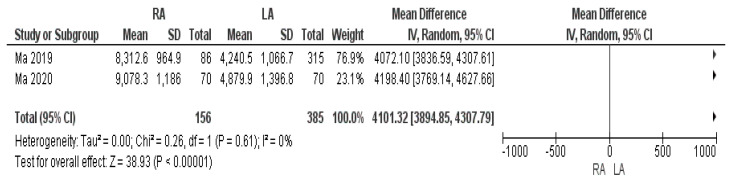
Cost of hospitalization [41,49].

Two studies assessed the total cost of hospitalization. The average total cost of the robotic procedure was considerably higher than laparoscopic surgery: USD 8695.45 and USD 4560.20, respectively. Our meta-analysis showed that this mean difference was statistically significant [MD USD 4101.32, (95% CI 3894.85, 4307.79) *p* < 0.00001], as shown in Figure 14.

## 4. Discussion

Laparoscopic adrenalectomy represents the gold standard treatment for benign adrenal tumors and, in selected cases, for small (diameter < 5 cm) malignant or suspected cortical carcinoma [2]. However, our review shows some of the advantages of the robotic approach over laparoscopic surgery and confirms the safety and feasibility of robotic adrenalectomy.

Our study revealed that RA is associated with a statistically significant lower mean intraoperative blood loss than LA, with a similar intraoperative RBC transfusion rate. Indeed, the robotic console facilitates more accurate organ dissection and hemostasis thanks to the greater field magnification and the more convenient ligation of blood vessels than the laparoscopic procedure [9,46,47].

Although it has been previously demonstrated that robotic surgery is more time-consuming than laparoscopy [33,45], our analysis showed a similar mean operating time between the two procedures: 157.2 min in the RA group and 149.6 min in the LA group. However, in order to confirm this finding and accurately compare results from different institutions, standardizing the collection of time data for the robotic procedure, such as the skin-to-skin time and/or docking and undocking times, is needed. Moreover, Piccoli et al. [7] and Pavan et al. (35) stated that the duration of the surgical procedure is influenced by both the side and the size of the adrenal mass, with longer operating times observed for right-sided RA compared to right-sided LA. However, no significant differences have been reported between the two approaches for left-sided tumors [7]. Furthermore, in the case of adrenal tumors ≤ 5.5 cm in size, Kim et al. [39] demonstrated a significantly shorter surgical time for LA over RA, and no differences in the duration of surgery were recorded for larger lesions between the two procedures. In addition, RA for large benign disease (nodule ≥ 6 cm) showed a shorter operating time than LA; however, no differences were observed between the two techniques in the case of malignant adrenal tumors [34]. Moreover, the learning curve (LC) is a critical metric for assessing the adoption of new surgical techniques in MIS, given its influence on surgical outcomes such as the operating time, blood loss and intraoperative complications. The learning curve for LA is well documented, requiring extensive experience to achieve mastery due to technical challenges such as its two-dimensional imaging, limited instrument mobility and ergonomics. Conversely, RA has been associated with a steeper but shorter learning curve due to its 3Dvisualization and enhanced dexterity. The robotic approach requires approximately 20 procedures to achieve proficiency compared to the extended learning period needed for LA. In detail, Agcaoglu et al. [28] reported a significant reduction in operative times after 10 RA, with a plateau reached after 20 procedures. Similarly, Brunaud et al. [25] reported a decrease in the operative time from 116 min to 87 min for RA with increasing surgical experience. Moreover, the introduction of robotic platforms has not only shortened the LC for experienced laparoscopic surgeons but also reduced inter-surgeon variability, particularly in anatomically challenging spaces like the retroperitoneum. Once the learning curve has been overcome, Kim et al. [39] demonstrated that the operative times for robotic posterior retroperitoneal adrenalectomy improved substantially, rendering the approach comparable to LA in terms of safety and clinical outcomes. Fang et al. [45] noted that experienced high-volume centers achieved lower conversion rates and shorter operative times with robotic surgery compared to laparoscopy. However, the benefits of robotics are tempered by the time required for set-up and the cost of the procedure, both of which could be progressively minimized with team experience and the standardization of protocols.

Emerging evidence suggests the potential superiority of the robotic system over laparoscopy, particularly in cases involving large tumors or obese patients. However, the surgical excision of large adrenal masses with MIS raises significant concerns, including accidental malignant tumor capsular disruption, partial mass removal, and the inherent difficulty of dissection in limited anatomical spaces, which increases the risk of local cancer recurrence. Therefore, further studies are needed to evaluate these perspectives [7,50].

The meta-analysis showed a slightly lower conversion to open-surgery rate in the robotic group than the laparoscopic group: 1.05% (11/1041) and 3.7% (85/2270), respectively; however, no differences in terms of the intraoperative complications rate emerged between the two groups. Previous abdominal surgery, extensive adhesions, a large tumor size, vascular involvement and the invasion of surrounding organs increase the complexity of both robotic and laparoscopic surgical procedures. Therefore, the well-known features of the robotic platform, such as tremor filtering, more precise dissection, endowristed instrumentation and 3D magnified vision, could explain the lower conversion rate registered in the RA group [33,43]. However, for large tumors (≥10 cm) or cases of suspected ACC, conversion to open surgery remains critical to ensure oncologic safety. As highlighted by recent studies, adherence to guideline recommendations advocating for conversion in complex cases is essential to reduce the risk of disease recurrence. In addition, surgical outcomes, particularly for malignancies, are strongly correlated with institutional and surgeon expertise. High-volume centers (>30 cases/year) and experienced surgeons (>20 cases/year) demonstrate superior adherence to oncologic principles. Therefore, the centralization of adrenal surgery in these centers is critical to ensure the safe implementation of MIS for complex cases.

Regarding the post-operative outcomes, our analysis found no differences in the overall complication rates, Clavien–Dindo ≥ III post-operative complications, re-admission to hospital, and 30-day mortality. No mortality was recorded in the robotic group. Therefore, it can be assumed that both RA and LA are relatively safe and reliable procedures for adrenal masses [35,42]. It is noteworthy that Piccoli et al. [7] demonstrated that both medical and surgical post-operative complication rates for robotic left-side adrenalectomy were significantly lower in the RA group than in the LA group. However, the two adrenal glands are located in two different anatomical districts, and left and right adrenalectomy should be considered as different operations. The right adrenal gland requires the mobilization of the right liver lobe with the vascular control of the inferior vena cava; on the other hand, the left adrenal gland involves the mobilization and detachment of the left colic flexure, the spleen and the distal tail of the pancreas. Therefore, further validations of these findings and a detailed comparative analysis of the two techniques for each of the two sides are needed.

Our analysis found no differences in the rates of R1 resection margins between the two approaches. However, the lack of stratification between benign, malignant, and metastatic tumors in the three studies included on this topic limits the oncologic interpretation of these findings. For malignancies such as ACC, where the resection margin has a significant impact on prognosis, achieving a R0 resection remains paramount. This underscores the need for careful patient selection and adherence to oncologic principles during MIS.

Interestingly, this meta-analysis revealed a statistically lower mean time of first flatus after surgery and a shorter length of hospital stay for the RA group than the LA group: 3 days and 4 days, respectively. Accordingly, it can be assumed that robotic surgery achieves faster oral re-feeding, patient recovery and discharge, potentially enhancing patients’ comfort and their quality of life after surgery. However, some authors previously demonstrated no significant differences in the length of hospitalization between the two strategies [9,39,45].

Our study revealed that the mean total hospitalization cost for robotic adrenalectomy is considerably higher than that for the laparoscopic approach: USD 8695.45 and USD 4560.20, respectively. This finding is consistent with previous reports in the literature [27,28,29]. However, the body of evidence supporting our result is weak due to the fact that only two of the included studies reported the total cost of hospitalization, which was defined as encompassing both intraoperative and postoperative expenditures, including time in the operating room, personnel expenses, and postoperative care [41,49]. Moreover, Agcaoglu et al. noted that the cost of additional robotic instruments and drapes, as well as the annual maintenance fees per procedure, may add up to around USD 900 to USD 950 per robotic procedure [27]. Nonetheless, the shorter hospital stay demonstrated for RA, the higher turnover of patients in high-volume referral centers, and the possibility of sharing robotic units with other surgical specialties could potentially reduce the overall costs associated with the robotic procedure and hospitalization [7]. Furthermore, as Feng et al. [40] stated, within an experienced surgical team, limits on the number of robotic instruments and energy devices employed keeps the total costs of both RA and LA comparable. However, the debate on the cost-effectiveness of robotic surgery remains unresolved, and further studies with standardized cost reporting are needed to assess whether these factors significantly lower the cost burden of the robotic system.

Our systematic review outlines most of the available evidence in comparing the outcomes of adrenalectomy between the laparoscopic and robotic approaches. To our knowledge, this is the largest and most recent meta-analysis that makes these comparisons.

It is worth noting that there are several limitations concerning this meta-analysis. Firstly, the surgical skills of surgeons were found to be non-uniform, and secondly, there was heterogeneity in the learning curves across the studies. For retrospective studies, variations in surgeon experience and the inclusion of cases within the early learning phase introduced potential biases that could confound comparisons between the two approaches. Furthermore, in many series, surgeons have already acquired proficiency in LA prior to transitioning to robotic surgery, which may potentially bias the LC results. The lack of standardized reporting on the exact number of cases required to reach proficiency across studies highlights a critical gap, necessitating the cautious interpretation of pooled outcomes. Moreover, the utilization of different surgical approaches (anterior versus posterior) and the heterogeneity in the size, laterality, and nature of the adrenal masses treated (most of the included articles did not report the final pathology exam) could contribute to incorrect final considerations. In particular, the lack of stratification between primary and metastatic tumors is a significant limitation of the present work, as the implications of the resection margin status across surgical approaches remain unclear, particularly for aggressive malignancies such as ACC.

In addition, the inclusion of small cohorts, particularly in relation to robotic procedures, in many studies could potentially limit the statistical power and lead to type II errors. Furthermore, the majority of studies were conducted in single-center settings, which may introduce institutional bias and limit generalizability. Most of the included studies were retrospective reports and with a study period potentially ranging from 1994 to 2019. Therefore, the variables analyzed exhibited intrinsic heterogeneity and structural bias, which could lead to misleading conclusions. Finally, according to the risk of bias assessment, while the majority of retrospective studies demonstrated high methodological quality with a low risk of bias in key domains, a subset exhibited moderate risks due to incomplete confounder adjustments and selective reporting, and one RCT presented minor concerns related to reporting and deviations from the intended interventions. These limitations underscore the heterogeneity of study designs and emphasize the need to interpret the findings with caution, particularly when integrating data from observational and randomized studies.

However, to partially handle these drawbacks, we always applied a random-effects model for the analysis. Further prospective randomized studies involving larger numbers of patients are therefore needed to understand which of the two methods is superior to the other in terms of oncological and surgical outcomes and the long-term results.

## 5. Conclusions

Laparoscopic adrenalectomy is considered the gold standard approach for the surgical management of benign adrenal tumors and, in selected cases, for small (diameter < 5 cm) malignant or suspected cortical carcinoma. However, robotic adrenalectomy has been demonstrated to be a safe and feasible procedure, with intraoperative and postoperative outcomes that are comparable to those of LA. Our meta-analysis suggests the slight superiority of RA over LA in terms of intraoperative blood loss, conversion to open surgery, time to first flatus, and length of hospital stay. Nevertheless, these conclusions should be interpreted with caution due to the inherent heterogeneity and biases of the included studies. In particular, the oncologic outcomes of MIS must be interpreted with caution, and adherence to guideline recommendations is essential to optimize patient safety. In the near future, RA could represent a promising complementary technique to LA, particularly in high-volume referral centers specializing in robotic surgery. However, its widespread adoption is currently limited by significantly higher costs. Further studies with detailed cost-effectiveness analyses alongside evaluations of the long-term outcomes are needed in order to more clearly delineate the clinical benefits of robotic adrenalectomy compared to the gold standard approach.

## Figures and Tables

**Table 1:** Summary of studies included in the meta-analysis.

Author	Region	Year	Study Period	Institution	Study Design	Sample Size		Surgical Approach	MINORS (Quality)
				Single or Multi		RA	LA		
Morino [48]	Italy	2004	2002–2002	Single center	RCT	10	10	ANT	NA
Brunaud [25]	France	2008	1996–2005	Single center	OCS (P)	50	59	ANT	22
Wu [5]	Taiwan	2008	2003–2005	Single center	OCS (P)	5	7	ANT	20
Agcaoglu [28]	USA	2012	2000–2011	Single center	OCS (P)	24	38	POST	22
Agcaoglu-2 [27]	USA	2012	2009–2011	Single center	OCS (P)	31	31	POST	23
Karabulut [29]	USA	2012	2008–2011	Single center	OCS (P)	50	50	ANT + POST	23
Aksoy [13]	USA	2013	2003–2012	Single center	OCS (P)	42	57	ANT + POST	23
Aliyev [30]	USA	2013	2008–2012	Single center	OCS (P)	25	40	ANT + POST	22
Dickson [31]	USA	2013	2009–2011	Single center	OCS (P)	23	23	POST	23
Pineda-Solis [32]	USA	2013	NA	Single center	OCS (R)	30	30	ANT	21
You [36]	Korea	2013	2009–2012	Single center	OCS (R)	15	8	ANT	22
Brandao [37]	USA	2014	2004–2013	Single center	OCS (P)	30	46	ANT	22
Raffaelli [38]	Italy	2014	1999–2012	Multi center	OCS (P)	13	5	ANT	22
Lairmore [33]	USA	2016	2005–2015	Single center	OCS (P)	17	76	POST	23
Morelli [16]	Italy	2016	1994–2014	Single center	OCS (R)	41	41	ANT	22
Pavan [35]	Italy	2016	2008–2013	Multi center	OCS (R)	80	337	ANT + POST	22
Colvin [9]	USA	2017	2000–2014	Single center	OCS (P)	20	16	ANT + POST	23
Feng [40]	USA	2018	2010–2017	Single center	OCS (R)	58	64	ANT + POST	22
Kim [39]	Korea	2019	2014–2017	Single center	OCS (R)	61	169	POST	22
Ma [41]	China	2019	2013–2018	Single center	OCS (R)	86	315	POST	23
Mishra [42]	USA	2019	2010–2013	Multi center	OCS (R)	51	238	NA	22
Niglio [43]	Italy	2019	2011–2018	Single center	OCS (R)	40	64	ANT	22
Smreen [44]	USA	2019	2009–2012	Multi center	OCS (R)	328	328	NA	22
Fang [45]	USA	2020	2000–2017	Multi center	OCS (R)	41	89	NA	22
Fu [46]	China	2020	2016–2019	Single center	OCS (R)	19	32	POST	23
Ma [49]	China	2020	2016–2019	Single center	RCT	70	70	ANT + POST	NA
Sforza [47]	Italy	2020	2008–2018	Multi center	OCS (R)	110	367	ANT + POST	22
Piccoli [7]	Italy	2021	2006–2019	Single center	OCS (R)	76	84	ANT	22

NA: not available; *OCS*: observational clinical study; P: prospectively collected data; R: retrospectively collected data; RCT: randomized controlled trial; ANT: anterior transperitoneal approach; POST: posterior retroperitoneal approach.

**Table 2:** General and patient characteristics.

	RA	LA	Studies (n)
Total patients included (n.) (*range*)	1446 (5–328)	2694 (5–367)	4140 (28)
Follow-up (months) (*range*)	28.3 (4.5–96)	38.6 (12–96)	7
Age (years) (*range*)	51.7 (38.5–62)	51.9 (40.3–60)	27
BMI (*range*)	28.3 (21.9–35.3)	29 (22.8–52.9)	26
Mean size of lesion (cm) (*range*)	4.17 (1.7–8)	4.01 (1.3–7.7)	26
Previous abdominal surgery (%)	201/607 (33.1%)	389/1166 (33.4%)	13
ASA score I/II (%)	208/374 (55.6%)	647/889 (72.8%)	7
ASA score ≥ III (%)	166/374 (44.4%)	242/889 (27.2%)	7
Side of lesion/surgery (n.)	961	1950	23
*Right* (%)	422 (43.9%)	924 (47.4%)	
*Left* (%)	523 (54.4%)	1005 (51.5%)	
*Bilateral* (%)	16 (1.7%)	21 (1.1%)	
Surgical approach (n.)	976	2039	25
*Anterior* (%)	683 (66.6%)	1040 (51%)	
*Posterior* (%)	343 (33.3%)	999 (49%)	
Tumor histology (n.)	1429	2618	27
*Malignant* (%)	132 (9.2%)	456 (17.4%)	
*Benign* (%)	1297 (90.8%)	2162 (82.6%)	

Continuous variables are expressed as mean; *ASA*: American Society of Anesthesiologists; BMI: body mass index; n: number.

**Table 3:** Technical and postoperative outcomes.

Surgical Outcome	Type of Surgery	Observations (n)	Mean or %	Studies Included (n)	*p* Value
Operating time (min)	RA	1067	157.2	26	0.06
	LA	2128	149.6		
Intraoperative blood loss (mL)	RA	779	66.9	18	0.04 *
	LA	1710	85.3		
RBC transfusion rate	RA	14/484	2.9%	8	0.98
	LA	35/1256	2.8%		
Conversion to open surgery rate	RA	11/1041	1.05%	25	0.007 *
	LA	85/2270	3.7%		
Intraoperative complication rate	RA	21/434	4.8%	10	0.29
	LA	41/1031	3.9%		
Time to first flatus (day)	RA	89	1.5	2	0.00001 *
	LA	89	2		
Overall complication rate	RA	101/1283	7.8%	24	0.96
	LA	184/2305	8%		
Clavien–Dindo ≥ III complication rate	RA	11/607	1.8%	12	0.45
	LA	16/1488	1.1%		
Length of hospital stay (days)	RA	956	3.06	25	0.00001 *
	LA	1952	4		
Readmission rate	RA	7/284	2.5%	5	0.39
	LA	17/811	2.1%		
R1 resection margin rate	RA	10/122	8.2%	3	0.87
	LA	41/373	11%		
30-day mortality rate	RA	0/582	0%	17	0.20
	LA	6/1032	0.6%		
Cost of hospitalization (USD)	RA	156	8695.45	2	0.00001 *
	LA	385	4560.20		

RBC: red blood cells; * *p*-value < 0.05: statistically significant result.

## Data Availability

Not applicable.

## Supplementary Materials

The following supporting information can be downloaded at: https://www.mdpi.com/article/10.3390/cancers17010150/s1, Supplementary File S1: PRISMA checklist [51], Supplementary File S2: Search strategy, Supplementary File S3: Funnel plots outcomes.
