# Supplementary material for: Robotic Versus Laparoscopic Adrenalectomy for Adrenal Tumors: An Up-to-Date Meta-Analysis on Perioperative Outcomes
Source: Cancers (Basel). 2025 Jan 5;17(1):150. doi: 10.3390/cancers17010150 (PMC11719468; doi:10.3390/cancers17010150)

Distribution of Risk of Bias Across Included Studies

The Newcastle-Ottawa Scale (NOS) was used to assess the methodological quality and risk of bias of the 26 included observational studies. Each study was evaluated across three primary domains: selection, comparability, and outcome, with a maximum possible score of 9 points [Table n.1 and Figures n. 1a].

Table n.1 - The Newcastle-Ottawa Scale scoring system

Study	Selection (max 4 points)	Comparability (max 2 points)	Outcome (max 3 points)	Total Score (max 9 points)	General Notes
Agcaoglu 2012	3	2	2	7	Some missing data on confounders
Agcaoglu-2 2012	3	2	3	8	Good follow-up, minor issues with selection
Aksoy 2013	4	2	3	9	Robust analysis; limited biases
Aliyev 2013	4	2	3	9	Strong methodology; detailed outcome tracking
Brandao 2014	4	2	3	9	Comprehensive reporting; detailed methodology
Brunaud 2008	4	2	3	9	Comprehensive analysis; well-controlled design
Colvin 2017	3	2	2	7	Some lack of detail in reporting confounders
Dickson 2013	4	2	3	9	Strong methodology; robust design
Fang 2020	4	2	3	9	Comprehensive study with good outcome analysis
Feng 2018	3	1	2	6	Focused on cost-analysis; lacks clinical outcomes details
Fu 2020	3	2	3	8	Clear reporting, good follow-up; minor selection issues
Karabulut 2012	3	2	2	7	Detailed outcomes but some missing data
Kim 2019	4	2	3	9	Robust analysis with extensive follow-up
Lairmore 2016	4	2	3	9	Comprehensive study with robust design
Ma 2019	4	2	3	9	Propensity score matching improves comparability
Mishra 2019	4	2	3	9	Strong methodology; clear reporting of outcomes
Morelli 2016	4	2	3	9	Well-controlled study with detailed data
Niglio 2019	4	1	3	8	Good reporting, limited control for confounders
Pavan 2016	4	2	3	9	Comprehensive study with global data; robust comparisons
Piccoli 2021	4	2	3	9	Detailed analysis with focus on laterality; well-reported outcomes
Pineda-Solis 2013	3	2	2	7	Good methodology but limited by sample size
Raffaelli 2014	4	2	3	9	Multicenter study; strong outcomes analysis
Samreen 2019	3	1	2	6	Focused on national database; limited individual-level data
Sforza 2020	4	2	3	9	Large international dataset; robust statistical analysis
Wu 2008	3	2	3	8	Early study; limited by technology at the time
You 2013	3	2	2	7	Single-surgeon study; limited generalizability

The majority of studies demonstrated a high methodological quality, with 73% (19 out of 26 studies) scoring 8 or above, indicative of low risk of bias. Only 11% (3 studies) received a score of 6 or below, highlighting moderate risk of bias in a small subset of the included studies. In term of "selection domain", the 85% of studies achieved 3 or 4 points, reflecting robust participant selection and ascertainment methods. In term of " comparability domain ", around 77% of studies (20 studies) received the full score of 2, indicating adequate control for confounders in the analysis. However, 23% (6 studies) showed limited adjustment for confounding variables, reducing their comparability. Then, in term of "outcome domain", the 88% of studies scored 2 or 3 points, reflecting accurate outcome assessment and sufficient follow-up periods.

Figure n.1a - The Newcastle-Ottawa Scale Risk distribution

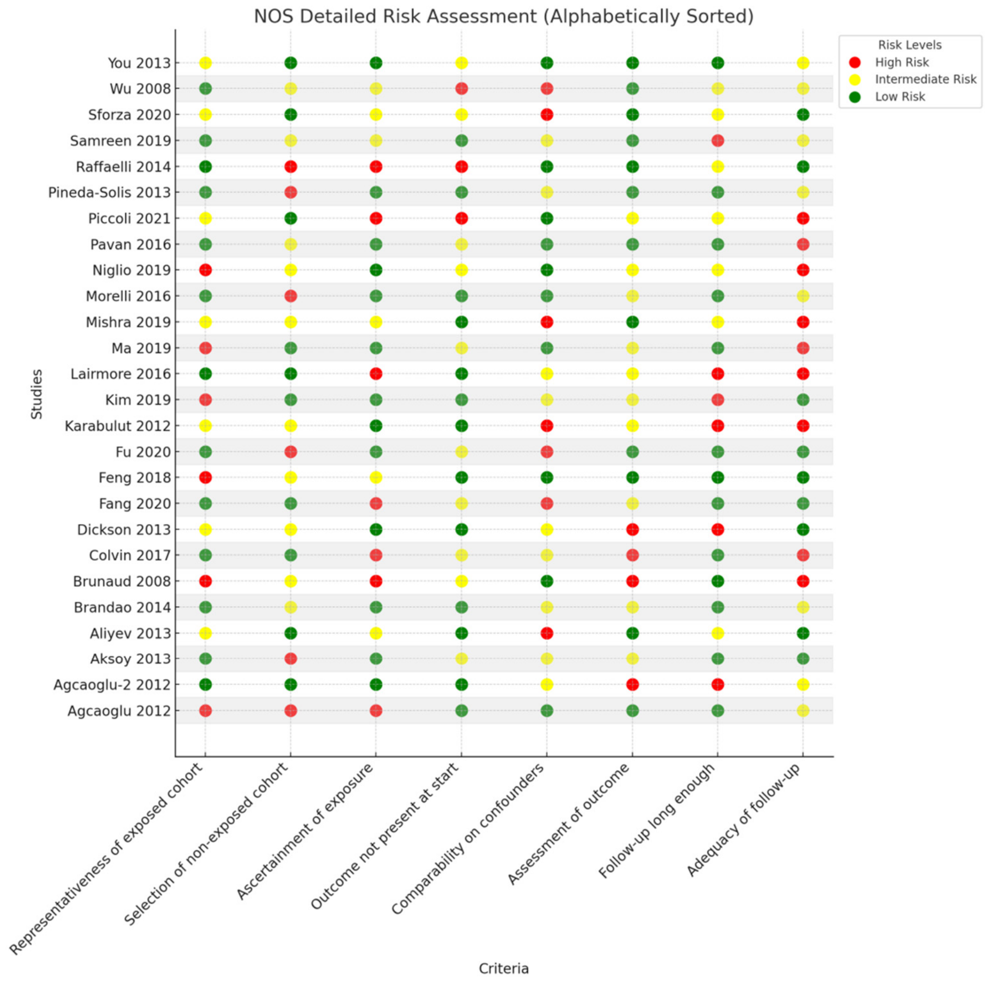

The majority of studies were categorized as low risk (scores 8-9), reflecting high methodological rigor and generalizability of findings. A smaller subset of studies showed moderate risk (scores 6-7), typically due to either incomplete follow-up data or insufficient control of confounding variables. A minimal fraction of included article fell into high risk category (scores ≤5), primarily due to deficiencies in selection procedures and lack of transparency in outcome assessment.

The Rob 2 tool was used to assess the methodological quality and risk of bias of the 2 included RCTs. Each study was evaluated across six primary domains: bias in randomization process, bias due to deviations from intended interventions, bias in measurement of the outcome, bias due to missing outcome data, bias in selection of the reported result and overall risk of bias [Table n.2 and Figure n.1b].

Table n.2 - The Rob 2 scoring system

Study	Bias in randomizatio n process	Bias due to deviations from intended interventions	Bias in measurement of the outcome	Bias due to missing outcome data	Bias in selection of the reported result	Overall Risk of Bias
Morino 2004	1 (Low)	2 (Some concerns)	1 (Low)	1 (Low)	2 (Some concerns)	2 (Some concerns)
Ma 2020	1 (Low)	1 (Low)	1 (Low)	1 (Low)	1 (Low)	1 (Low)

Figure n.1b - The Rob 2 system distribution

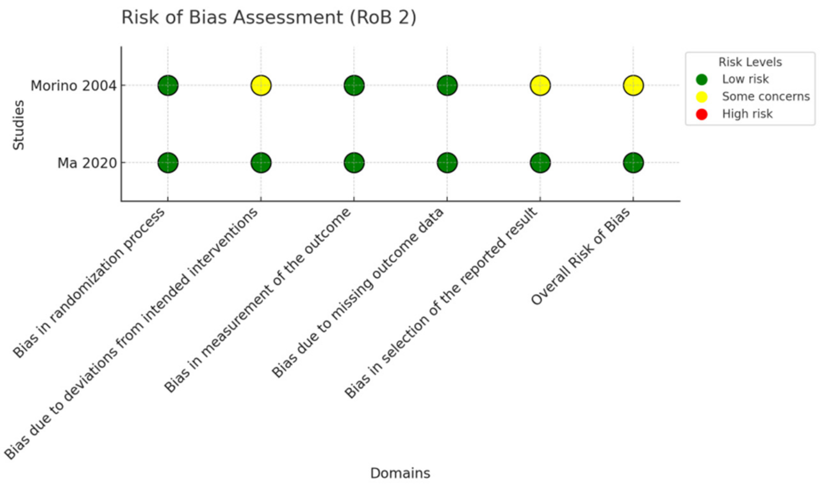

Ma 2020 et al. showed a low risk of bias across all domains. The use of randomization, robust blinding, comprehensive outcome measurement, and the absence of missing data highlight its methodological rigor. This study represents a strong addition to the meta-analysis. On the other hand*, Morino 2004 et al.* exhibited some concerns in two domains of the RoB 2 assessment: bias due to deviations from intended interventions and bias in selection of the reported result. Indeed, this study lacks detailed reporting on whether blinding was implemented for participants and personnel. While this study appears methodologically robust, the absence of a pre-specified protocol raises concerns regarding selective reporting of outcomes. This limits confidence in the reliability of the reported results. Despite these limitations, *Morino 2004 et al.* demonstrates low risk of bias in critical domains, such as the randomization process, measurement of outcomes, and handling of missing data, ensuring a generally reliable foundation for inclusion in a meta-analysis. However, together, these 2 studies provide complementary insights and are sufficiently robust to contribute meaningfully to the meta-analysis.

Conclusion

The 26 observational studies included in the present meta-analysis are predominantly of high quality, with well-distributed low risks of bias across domains. This strong methodological foundation supports the reliability of the meta-analytic findings, though caution should be exercised in interpreting results influenced by studies with moderate or high risk. Among the 2 RCTs, one trial presented minor concerns related to reporting and deviations from intended interventions, while the other demonstrated a consistently low risk of bias. These limitations highlight the heterogeneity in study designs and underscore the need for cautious interpretation of findings, particularly when integrating data from observational and randomized studies.

The publication bias of the included studies was tested and the Funnel plots for each outcome are provided in Figures.

Figure n.2 - Operating time (min)

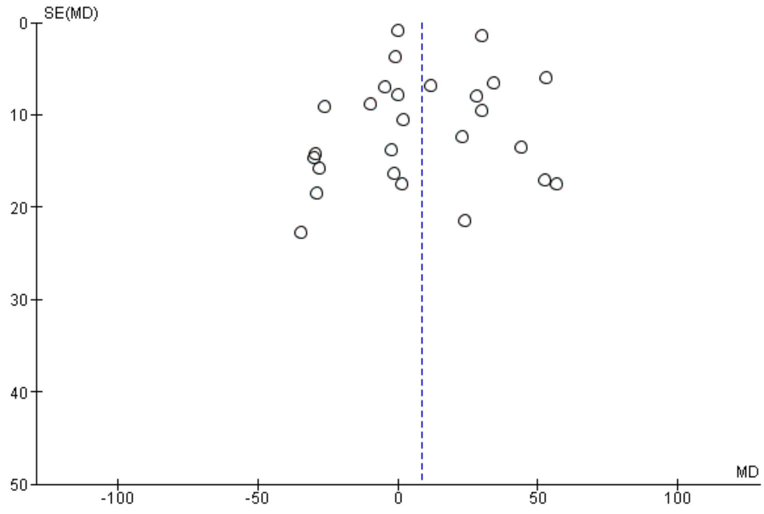

Figure n.3 - Intraoperative blood loss (ml)

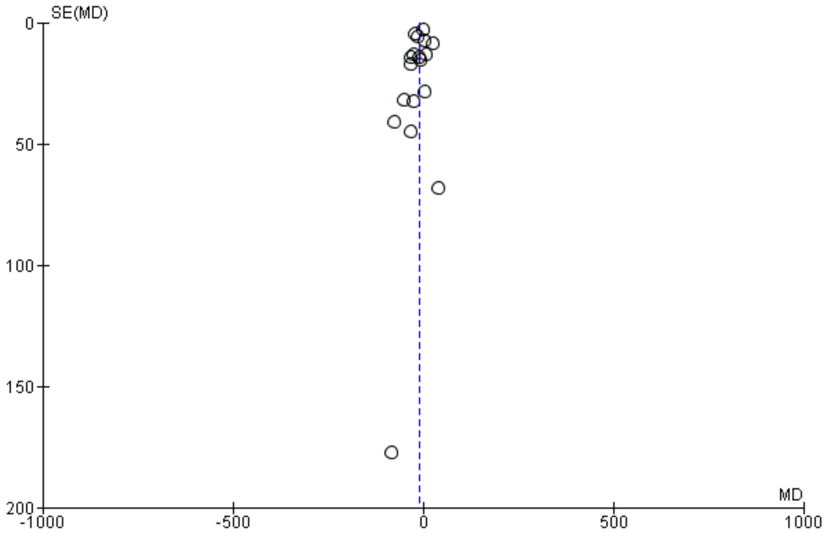

Figure n.4 - Intraoperative Red Blood Cell (RBC) transfusion rate

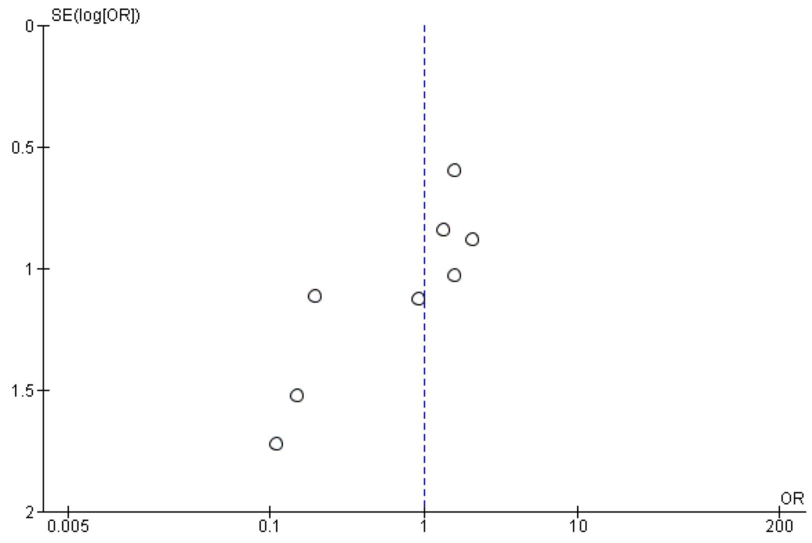

Figure n.5 - Conversion to open surgery rate

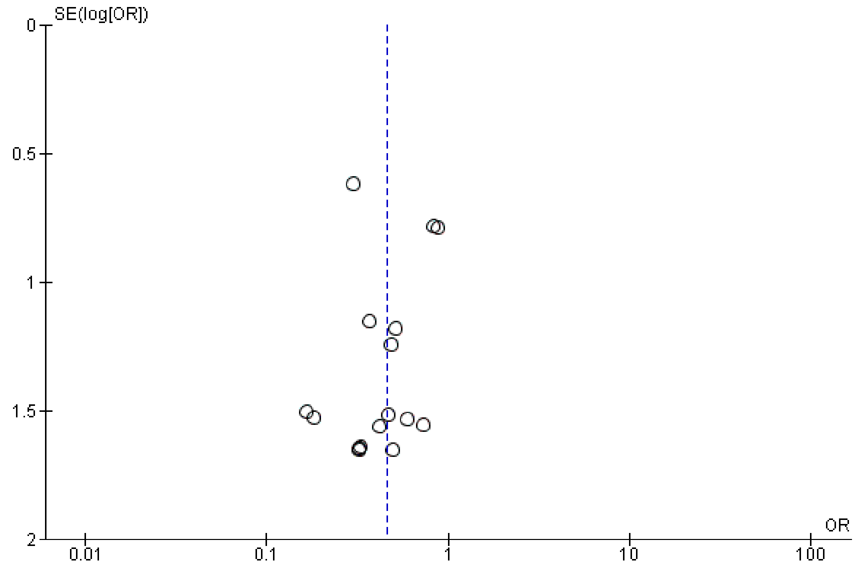

Figure n.6 - Intraoperative complication rate

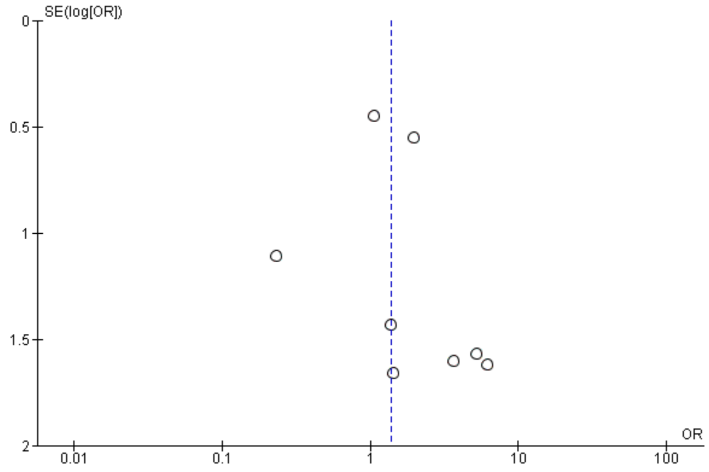

Figure n.7 - Time to first flatus

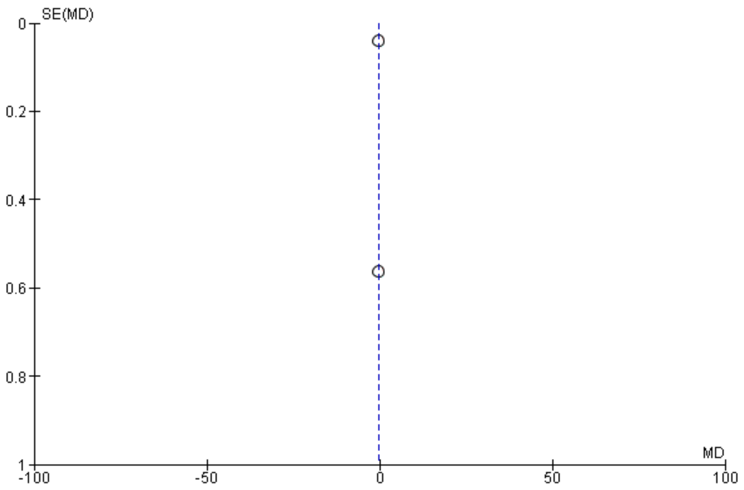

Figure n.8 - Overall complicationrate

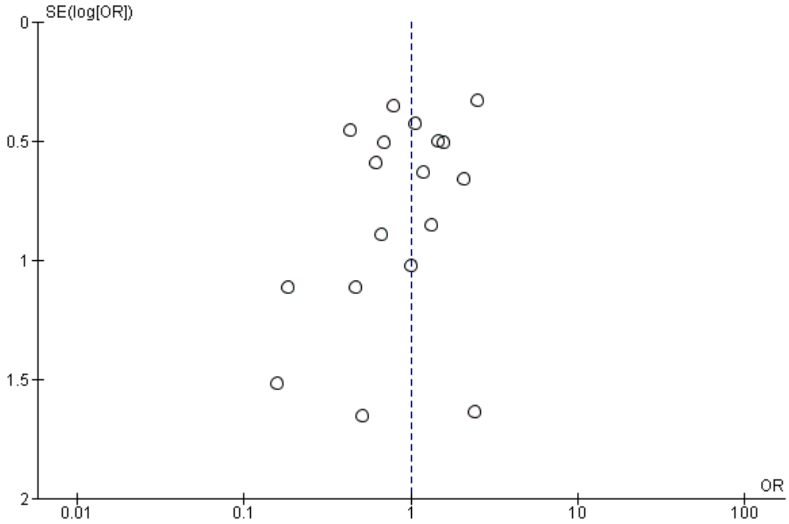

Figure n.9 - Clavin-Dindo ≥ III complication rate

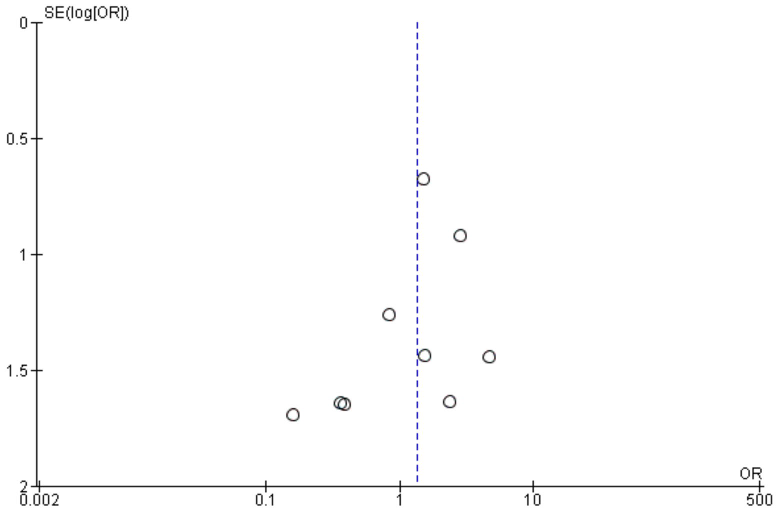

Figure n.10 - Length of hospital stay

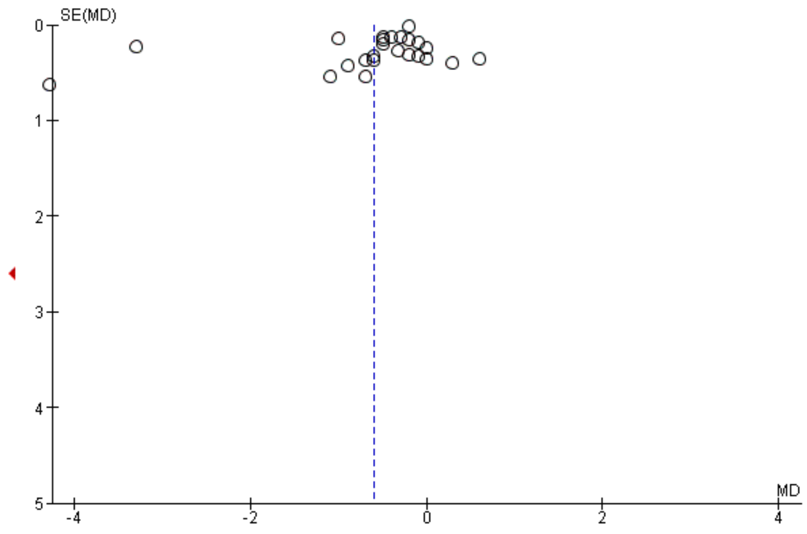

Figure n.11 - Readmission rate

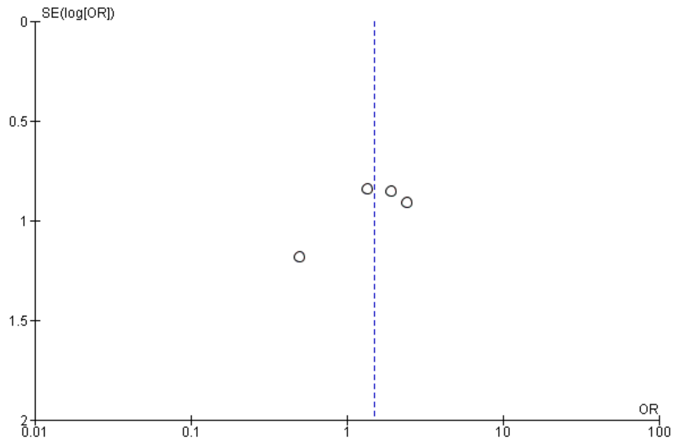

Figure n.12 - R1 resection margin rate

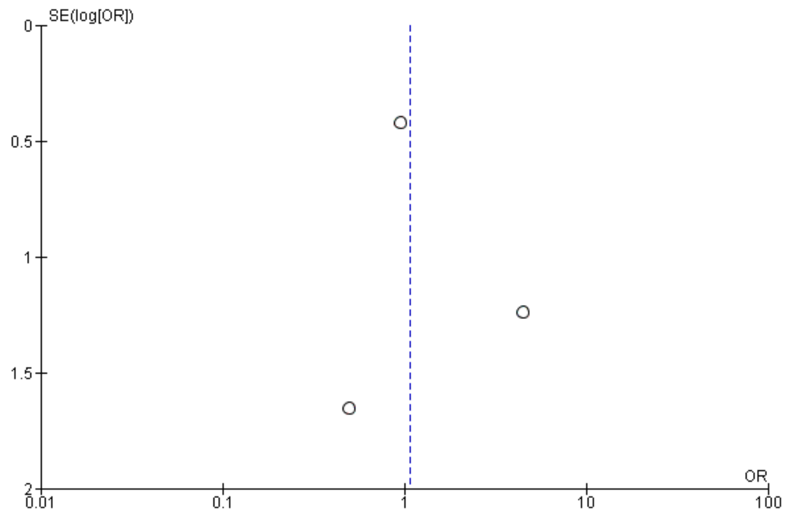

Figure n.13- 30-day mortality rate

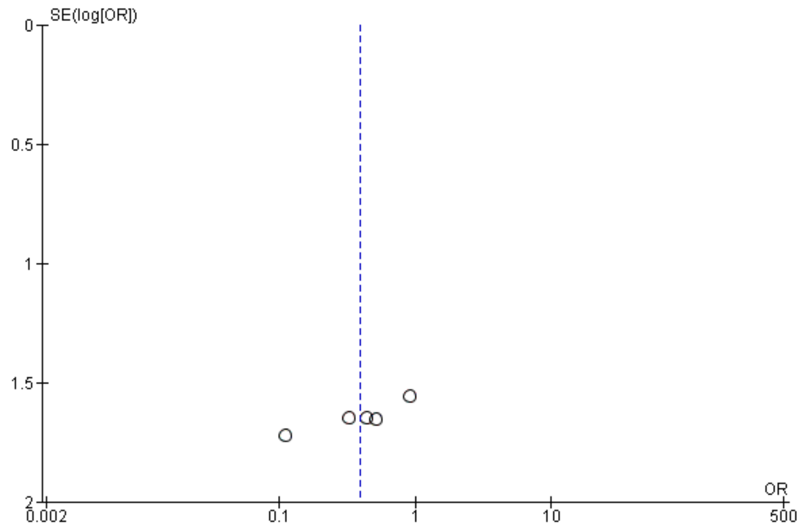

Figure n.14 - Cost

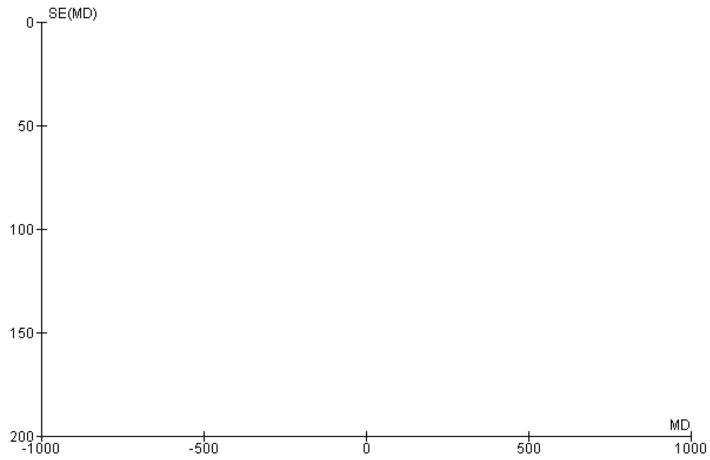

Supplement: Supplementary file 1 [file cancers-17-00150-s001.zip › Supplementary file S3.pdf]
